# In-situ simulations for COVID-19: a safety II approach towards resilient performance

**DOI:** 10.1186/s41077-020-00137-x

**Published:** 2020-07-29

**Authors:** Zavi Lakissian, Rami Sabouneh, Rida Zeineddine, Joe Fayad, Rim Banat, Rana Sharara-Chami

**Affiliations:** 1 Dar Al-Wafaa Simulation in Medicine (DAWSIM), American University of Beirut Medical Center, Beirut, Lebanon; 2 Department of Pediatrics and Adolescent Medicine, American University of Beirut Medical Center, P.O. Box 11-0236 Riad El Solh, Beirut, 110 72020 Lebanon; 3 Department of Fine Arts, American University of Beirut, Beirut, Lebanon

**Keywords:** COVID-19, In situ simulation, Safety I, Safety II, Latent safety threats, Multidisciplinary teams, Hospital preparedness, Quality improvement

## Abstract

**Background:**

COVID-19 has taken the world by surprise; even the most sophisticated healthcare systems have been unable to cope with the volume of patients and lack of resources. Yet the gradual spread of the virus in Lebanon has allowed healthcare facilities critical time to prepare. Simulation is the most practical avenue not only for preparing the staff but also for troubleshooting system’s latent safety threats (LSTs) and for understanding these challenges via Hollnagel’s safety I–II approaches.

**Methods:**

This is a quality improvement initiative: daily in situ simulations were conducted across various departments at the American University of Beirut Medical Center (AUBMC), a tertiary medical care center in Beirut, Lebanon. These simulations took place in the hospital with native multidisciplinary teams of 3–5 members followed by debriefing with good judgment using the modified PEARLS (Promoting Excellence and Reflective Learning in Simulation) for systems integration. All participants completed the simulation effectiveness tool (SET-M) to assess the simulation. Debriefings were analyzed qualitatively for content based on the Safety Model and LST identification, and the SET-Ms were analyzed quantitatively.

**Results:**

Twenty-two simulations have been conducted with 131 participants. SET-M results showed that the majority (78–87%) strongly agreed to the effectiveness of the intervention. We were able to glean several clinical and human factor safety I–II components and LSTs such as overall lack of preparedness and awareness of donning/doffing of personal protective equipment (PPE), delayed response time, lack of experience in rapid sequence intubation, inability to timely and effectively assign roles, and lack of situational awareness. On the other hand, teams quickly recognized the patient’s clinical status and often communicated effectively.

**Conclusion:**

This intervention allowed us to detect previously unrecognized LSTs, prepare our personnel, and offer crucial practical hands-on experience for an unprecedented healthcare crisis.

## Background

The Washington Post recently reported that “somehow, this messed-up country [Lebanon], teetering on the brink of economic ruin and political chaos, has done something right when it comes to the coronavirus.” [1]. How has a country amid crippling protests since October 2019, soaring food prices, lack of healthcare resources, and a currency in free-fall managed to nearly plateau the COVID-19 curve? The latest COVID-19 numbers for Lebanon show 1466 confirmed cases and 32 deaths [2]. Comparatively, other countries with similar populations (6 million) such as Singapore (> 40,800 cases) and Norway (> 8600 cases) have not fared as well [3].

People did not trust the Lebanese government would take the necessary measures to control the spread of the virus, so they were proactive in taking extreme precautions from early on—the government soon followed by implementing strict curfews and closing down public gathering spaces. In addition, many had already begun working from home during the 2019 protests or had lost their jobs and were more likely to be home already. Therefore, the gradual spread of the infection in Lebanon allowed healthcare facilities time to prepare and expand hospital capacity, to the point that there are now more beds available than patients to fill them [4].

One of the most practical ways to help hospitals and healthcare personnel have prepared for the pandemic is simulation [5–7] as has been shown during previous healthcare crises such as severe acute respiratory syndrome (SARS), Ebola, and influenza A [8–11]. Healthcare is already a high-risk, high-stress setting prone to errors, even more so when the stakes are higher for both patients and the healthcare professionals themselves and when the guidelines are uncertain [12]. Therefore, it is imperative that medical teams have the opportunity to familiarize themselves with potential clinical scenarios, be situationally aware and cognizant of their environment, and demonstrate effective teamwork behavior by practicing key crisis resource management elements including closed-loop clear communication, distribution of workload, efficient role assignment, and setting priorities dynamically [13, 14]. Alongside training personnel, simulation can also be useful for troubleshooting the system to discover latent safety threats which may have gone unrecognized and proven devastating to patient safety [15].

Institutions around the world have shifted their perceptions of simulation as a “backburner” training tool to a “first choice” strategy for ensuring staff and system readiness in the face of COVID-19 [16], yet we cannot disregard the practical constraints of performing simulations, especially in situ during the pandemic, such as the need for physical distancing, rigorous infection control for the simulators, the equipment, and the participants [17]. Within this dichotomy, we are given the opportunity to assess the staff’s and the system’s flexibility to adapt their day-to-day activities to suit the uncertainties caused by the COVID-19 pandemic. Most would define safety as the absence of accidents and/or incidents, assuring minimal to acceptable risk to the patient. Hollnagel describes two approaches to analyzing safety in healthcare: safety I presumes that if an incident were to occur, then it is due to clear and identifiable failures or malfunctions of technology, procedures, personnel, or the organization; these threats must be identified and either eliminated or resolved [18]. However, given the uncertainty and complexity of healthcare work, the surprise is not that things occasionally go wrong but that they actually go right more often. Therefore, instead of focusing on what went wrong, perhaps we should start looking at factors which contribute to successful outcomes—this is the safety II perspective discovering the system’s adaptability to varying conditions [19], focusing on exploring how successful performances are produced via adaptive mechanisms on the part of personnel or the system itself in the midst of uncertainty [20]. According to safety II, the reason why things go right is the performance variability observed every day [work-as-done] in order to respond to complex, challenging, and varying conditions [21]. Therefore, prior to the implementation of change, determining how success is achieved normally (work-as-done) should focus not only on best practice but also on the various adjustments and trade-offs healthcare workers make to achieve success under the conditions they face, including where resources are limited. The implication is that when flexibility is proven successful, protocols should allow variability [22].

As Patterson et al. explain, surprise is inevitable in healthcare, yet the “ability to recover from that surprise depends on what capacities are already present that can be deployed to address the unexpected” (p. 70, 2019) [23]. In situ simulation—though controlled—offers the perfect opportunity to test permutations of surprises coming as close to “work-as-done” without compromising actual patient safety [23]. Although we had not simulated infection control scenarios in the past, we believe technical and non-technical skills gained from pre-COVID-19 simulations would reflect in the management of COVID-19 simulated patients. Moreover, as Hollnagel describes it, the basis of resilient performance in healthcare [24–28] is the ability to respond, monitor, learn, and anticipate—these are transferable abilities towards a safety II management approach [25].

In an effort to prepare for the challenges of COVID-19, we adopted Hollnagel’s Safety Model to glean latent safety threats by simulating COVID-19 scenarios in various departments within our academic tertiary hospital.

## Methods

### Aim, design, and setting

This is an ongoing hospital-wide quality improvement initiative for multidisciplinary team preparedness for COVID-19 and the identification and pre-emption of latent safety threats. The intervention is taking place at the American University of Beirut Medical Center, an academic tertiary healthcare facility located in urban Beirut, Lebanon. In situ simulations occur in the newly established COVID-19 intensive care units (ICUs) and ward, the adult and pediatric ICUs and wards, the emergency department, and the labor and delivery suite. The locations are chosen in collaboration with the simulation champions and faculty of the relevant department and based on the availability of space, staff, and patient occupancy.

### Characteristics of participants and description of materials

Multidisciplinary teams including attendings, residents, fellows, nurses, students, and consultants such as respiratory therapists are included in the intervention. Simulations usually begin with a core team of 2 residents and 1 or 2 nurses; attendings, fellows, and respiratory therapists are called in during the scenario on a needs basis. Participation and attendance per simulation, including the observers, the simulation technologist, and the debriefers, do not exceed ten.

**Fig. 1:**
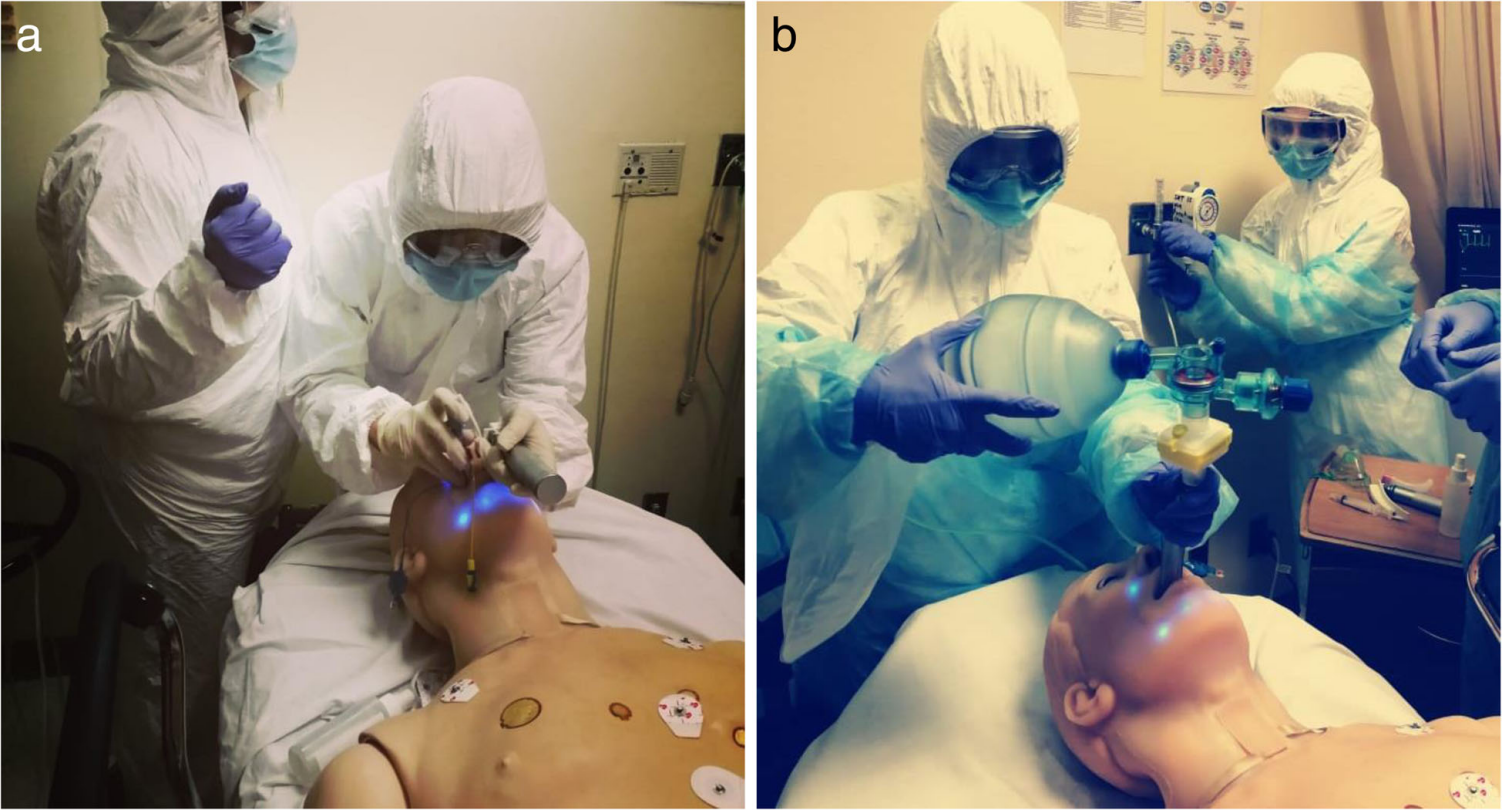
Residents in full PPE, working on HAL in the a emergency department and b PICU (All images are the property of DAWSIM, AUBMC. They were taken and are shared by the consent of those in the pictures.)

**Fig. 2:**
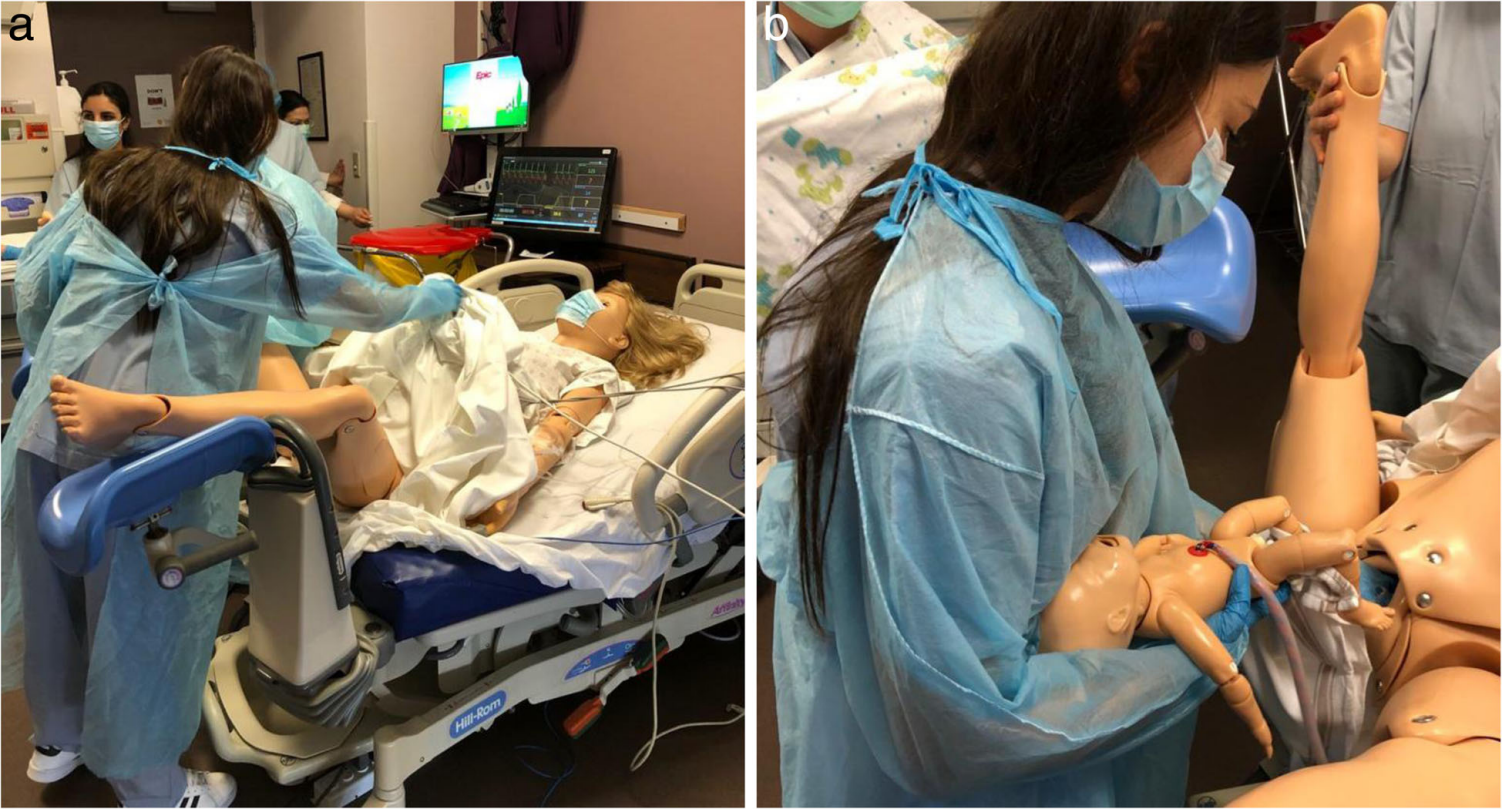
**a** Residents working on NOELLE® and **b** OMNI® in the labor and delivery suite (All images are the property of DAWSIM, AUBMC. They were taken and are shared by the consent of those in the pictures.)

Based on the targeted team and location of the simulation, one of the following high-fidelity simulators is used:

1. HAL® Advanced Multipurpose Tetherless Patient Simulator (Gaumard Scientific, Miami, FL, USA). As tetherless simulator technology (TST), HAL® operates continuously and wirelessly, allowing for training in the working environments and during transportation. Moreover, it is receptive to real resuscitation equipment and drug recognition and can transition between physiologic states in response to commands and interventions [29]. In appearance, HAL® is an adult male; therefore, he is mostly used in the adult ICUs/wards and in pediatrics when simulating a teenager (Fig. 1). The COVID-19 scenario was adapted from Laerdal Medical on infection prevention and control (IPC): severe acute respiratory infection [Novel COVID-19 SARI] [30].

2. PediaSIM® (CAE Healthcare, 2017) provides advanced pediatric simulation training; therefore, it is used in the pediatric ICU/wards and the emergency department, so healthcare providers can improve team performance and communication in pediatric critical care. PediaSIM® represents a 6-year-old patient with a comprehensive set of clinical features for trauma, nursing, and emergency response. Learners can practice and achieve mastery in a range of pediatric critical interventions, including needle cricothyrotomy, chest tube insertion, and airway management [31].
3. NOELLE® S550—the maternal care patient simulator with OMNI® (Gaumard Scientific, Miami, FL, USA). The NOELLE S550 Maternal and Neonatal Birthing Simulator is designed to provide a complete birthing simulation experience before, during, and after delivery [32]. This manikin was used to simulate a COVID-19-positive woman in labor; the scenario was adapted from the Society for Obstetric Anesthesia and Perinatology [33] (Fig. 2).

### Description of processes and interventions

Simulations are coordinated and scheduled with each department’s simulation champion a week in advance and confirmed on the day of to guarantee availability of space, staff, and no patient occupancy. The teams are assembled from personnel already on the unit with the assistance of the chief resident and the nurse manager, both of whom cover the participants’ duties during the intervention. The simulation technologist pre-briefs the participants and orients them to the simulator prior to starting the scenario. Given the general PPE shortage, we have secured several sets of gowns, goggles, and shields which are being disinfected and reused solely for simulation purposes, and participants are always required to have their own masks. The participants are then given the case history and the scenario begins. This portion usually lasts 15–20 min on average and is immediately followed up by an expert facilitated debriefing “with good judgment” [34] using the modified PEARLS for systems integration [35]. The debriefing is done at the bedside and lasts 30–40 min.

Following the simulation, participants complete the SET-M based on a 20-item 3-point ordinal scale [3—strongly agree, 2—somewhat agree, 3—do not agree] instrument to assess the effectiveness of the pre-briefing, the simulation scenario, and the debriefing in achieving the learning objectives for the activity [36].

All simulation activities [pre-briefing-scenario-debriefing] are audio and video recorded.

#### Analysis

The SET-Ms were analyzed quantitatively using IBM SPSS 23; descriptive analyses were performed on categorical, ordinal data to derive percentages. Debriefings were transcribed, and the content was reviewed for clinical incidences and participant behavior based on Hollnagel’s safety I–II model; LSTs were gleaned from safety I.

## Results

So far, twenty-two simulations have been conducted with 131 active participants. SET-M results show that most participants strongly agree that the simulation improved their knowledge and confidence of both clinical and efficient teamwork skills (Additional File 1).

Latent safety threats were derived from incidents classified as safety I, and we reviewed the debriefings and organized these based on clinical and human factor issues (Table 1). Clinical incidents pertained to (1) incorrect donning and doffing of the PPE, which led to safety hazards to both the patient and the healthcare staff; (2) oxygenation: whether it was the use of non-rebreather masks at high flow or bag mask ventilation outside negative pressure rooms, the staff’s lack of knowledge of the changing guidelines with regard to the appropriate choice of oxygen supplementation increased the chances of viral aerosolization; (3) general unfamiliarity and experience with intubation and specifically lack of knowledge of rapid sequence intubation (RSI) procedures and guidelines, participants were unfamiliar with the equipment needed for RSI and the proper sequence of connections, and they used the wrong dosage for the medications leading to delayed intubation, viral aerosolization, and increased risk of aspiration; and lastly, (4) overcrowding the room and wheeling the emergency cart into the room and thus adding to the risk of contamination.

**Table 1:** The Safety Model on clinical aspects and crisis resource management

Department	Safety I	Latent safety threat identified	Observed participant behavior	Recommended solutions	Observed improvements	Explanations of good performance
**Clinical aspects**						
PICU, ICU	-PPE donning/doffing technique	-Inadequate staff preparedness in infection control	-Inexperience in donning/doffing PPE -Delay in donning and doffing PPE -Multiple contamination hazards due to lack of prior training in PPE	-Donning and doffing posters at room entrances and exits -Colleagues in the room assist in donning/doffing PPE -Sharing videos of proper donning/doffing technique -Simulations for practicing donning/doffing	-Recognizing the need for additional PPE	-The infection control team and the simulation team led the effort throughout the hospital on the correct donning/doffing protocols. -Donning/doffing videos were produced and e-mailed to the staff; posters were placed on every door to remind healthcare workers of the proper techniques. This not only helped healthcare workers, but patients/families as well.
	-Using non-rebreather mask at a high flow -bag-mask ventilation -Using non-invasive ventilation outside negative pressure rooms	-Increasing the chances of viral aerosolization	-Lack of knowledge of aerosol generating procedures	-Guideline adjustments to include warnings about aerosol generating procedures -Simulations targeting oxygen supplementation	-Choosing non-rebreather facemask as first line oxygen supplementation -Use of MDI instead of nebulizers -Identifying respiratory failure signs	-Participants exhibited adequate knowledge in regard to respiratory therapy due to knowledge dissemination by intensivists and subsequent attendance of skill-specific simulation part-task training sessions organized by the simulation program. -In pre-COVID simulations, timely and accurate oxygenation had been prioritized, participants were aware of the needs and methods, however, were slow in adapting these needs to the nuances of COVID-19 patients.
	-Aggressive fluid resuscitation in case of shock	-Risk of fluid overload	-Lack of knowledge of fluids restriction guidelines	-Simulations targeting management of shock	-Early inotropic support	-Pre-COVID simulations stressed on the importance of recognizing low cardiac output and the need for early ionotropic support. -Displaying adherence to the “Precautionary Principle”
	-Inexperience in intubation -Unfamiliar with intubation equipment -Improper rapid sequence intubation (RSI) medication doses -Unfamiliar with proper sequence of connections	-Delay in intubation -Intubation procedure interruptions -Aerosolization of viral particles -Increased risk of aspiration	-Uncomfortable with intubation and its equipment -Unfamiliar with detailed RSI concepts	-RSI training sessions -Simulations targeting intubation	-Recognizing the need for RSI -Proper RSI medications -Not using bag-mask ventilation for pre-oxygenation	-Skills-specific RSI sessions were conducted throughout the hospital and for all residents by the simulation team. When faced with a COVID-19 scenario, residents recognized the need for RSI and followed the new recommendations.
	-Presence of unnecessary staff in the room -Getting the emergency cart inside the room -In the L&D suite: small space when the infant incubator was placed in the room	-Increase risk of contagion and contamination	-Contamination hazards due to lack of knowledge of proper infection control measures	-Guidance on proper role assignment and environment control -Reorganizing the environment to distance the labor bed from the infant incubator by removing unnecessary furniture from the room	-Preparing necessary medications and equipment outside the room -Obs. team tried their best to avoid contamination -Timely arrival of neonatal COVID team to the room	-Multiple sessions provided by infection control on a weekly basis communicated to the staff the hazards of overcrowding in rooms, the importance of following prevention and contamination protocols. These elements were also alluded to during pre-briefing. -Over time, hospital staff became familiar and comfortable with simulation, calls were not disregarded, and consults arrived to the simulations in a timely manner.
**CRM**						
PICU	-Disorganization due to lack of leadership and proper role assignment	A fraught atmosphere	-Residents felt stressed, confused and disorganized during the simulation R: Stressful. Very stressful.” “R: We weren’t organized. Multiple people were giving orders at the same time. We should have asked who the leader was, who was making the decisions. -N: It was confusing at first because there was not a clear leader. I did not know who to listen to.”	-Longer pre-briefings, allowing the participants to truly get comfortable in the simulated environment Simulations and subsequent debriefings focused on non-technical skills and teamwork emphasizing timely role assignment, closed-loop communication and sharing mental models -Build a culture of teamwork, flatten the hierarchy, encourage personnel to step up, take charge and speak up without fear -Developing an equipment check list for a more timely and efficient management of resources. Implementing skills training in situ, e.g., CPR in a patient room to familiarize personnel with the positioning of the bed, the location of the board, etc. -Send expecting parents a detailed description of what to expect ahead of labor and follow institutional guidelines	-Closed-loop communication	-This is the result of having participated in multiple high-fidelity simulations in the past and working in multidisciplinary teams. This was particularly helpful in COVID-19 simulations because healthcare workers from different teams were not always necessarily familiar with working together.
		-Inadequate allocation and management of human resources	-At times there were more personnel in the room than needed “R: So many faces! There should not have been that many people. Only essential personnel.”			
		-Hierarchical culture, failure to take charge/lead	-The initial leader often took a step back after a senior physician came in, and allowed them to take control without clearly vocalizing and reassigning the role. “F: When I entered I noticed that they were a bit lost and confused so since I am the fellow I decided to take over, but I did not say that I just assumed that leadership was handed to me”			
ICU	-Slow, inefficient response	-Lack of situational awareness	-Residents had difficulty locating and setting up the needed equipment, which delayed their response. -“R: The problem was that we could not find the needed medication on time. The key is to find the proper response at the proper time. That was the stressful part” Residents did not take the necessary measures which would have facilitated their delivery of care such as lowering side rails, using CPR board or lowering the bed -“D: Why weren’t you comfortable doing the CPR? R: Because of our positioning. It was uncomfortable. D: How could you have made it more comfortable for yourself? - R: Maybe lowering or better positioning the bed. We also forgot the board.”	-Residents often rely of nursing staff to be situationally aware of the location of medications, equipment, etc., and in critical instances when they ought to fend for themselves, they are lost. It is imperative that everyone is familiar with the location of the medical carts on the floors, which are identically equipped, and this must be included in the orientation week of all new personnel and refreshers should be given every 6 months. -Often resuscitation or intubation trainings take place on part-task trainers, either places on tables or the floor; therefore, personnel are not as familiar with the most comfortable positioning of the bed when performing life saving measures on actual patients. We propose trainings using the patient bed, and the mechanisms of lowering the rails, the bed, positioning of the board, etc.	-Good role assignment	-This is also is the result of having participated in multiple simulations in the past, prior to this pandemic. So, leaders properly assigned roles and tasks according to the skills, experience, and comfort of available personnel. He/she gave every team member in the room a clear role, reminded them of it in case they got distracted doing something else, and re-shuffled when necessary.
Obstetrics (L&D)	-Unfamiliarity with in situ simulation in the labor and delivery suite -Disorganization in a small room when the pediatric team arrived -Poor communication with the mother as to what to expect when baby was born	-Need for more hospital-wide multidisciplinary simulation exercises	-During the debriefing session, participants expressed how important and beneficial the hands-on practical aspect of the simulation was. -“R: I feel like this was very important for everyone. It shows you what you need to work on and reminds you of the information that you need to remember. Really every one of us should go through this. Now I feel like I know and understand the guidelines more -R: Everyone should participate in one. It would be good if we can repeat this. Can we do another one next week?”		-Openness and willingness to learn and improve	-Participants acknowledged the shortcomings of their performance and the areas they need to work on. They were quick to ask for help especially that the system supports seeking guidance from seniors or more experienced health providers with no judgment or jeopardy. They showed an aptitude for constructive criticism and readiness to learn and improve their delivery of care in future cases. Being able to admit the times when one is at fault is in itself a positive notion that can be placed under the umbrella of safety II

As we repeated these simulations, we noticed the adaptability of the participants’ behavior as they learned new skills and acquired new knowledge, which eventually led us to explore a safety II perspective: (1) participants were quick to recognize and don their PPE, (2) they were able to identify the signs of respiratory failure and the need for non-rebreather facemasks as their first line of oxygen supplementation and metered-dose inhalers (MDIs) instead of nebulizers for inhaled treatments, (3) they initiated early inotropic support for shock instead of aggressive fluid resuscitation, (4) they improved on RSI procedures and medications dosing, and (5) they controlled the level of contamination by preparing medications and equipment outside the room.

The clinical incidents did not occur in vacuum, and they were often due to non-technical human errors such as (1) disorganization due to poor leadership and ineffective role assignment leading to (2) slow and inefficient response. However, participants were often comfortable with closed-loop communication, especially when the hierarchy was flatter. They became more flustered however when a fellow or an attending was called in and the leadership instinctively shifted over without explicit reassignment of roles. The latter is a cultural phenomenon in countries such as Lebanon [37], where control is automatically ceded to the most senior person in the room.

## Discussion

The COVID-19 pandemic has posed a challenge even for the most sophisticated healthcare systems. Given the unprecedented scale and rate by which it has spread, there was no way of fully anticipating and preparing for all eventualities. It comes as no surprise that simulating such a rare clinical crisis has proven to be highly effective in raising our participants’ confidence and improving their clinical and non-technical skills. Importantly, this intensive training has also allowed us to discover latent safety threats which will be useful well beyond the duration of the COVID-19 pandemic. As we started these simulations, the focus was more on the clinical approach to COVID-19 patients because of the new knowledge that had to be acquired and because of some deviations from practice of non-COVID care. Naturally, there were many gaps to address from a safety I approach. This led to intensive specific skills training on rapid sequence intubation and cardiac arrest in COVID-19 patients that were not part of the high-fidelity simulation scenarios. This deliberate practice has proven of utmost importance especially when addressing rare events and has complemented reaching saturation in clinical competency during simulations. Looking through the safety II model lens, and when it comes to non-technical skills, we believe that we reaped many of the rewards of our previous simulation activities. Pre-COVID-19, we routinely performed in situ simulations and mock codes tailored to challenge participants’ flexibility and force them to adapt to unpredictable events, whether pertaining to the simulated patient’s clinical status or systemic obstacles in order to deliver efficient, timely, and effective care, keeping Hollnagel’s four basic abilities for resilient performance in mind. Participants’ increased confidence and familiarity presented themselves as key building blocks for the performance improvement observed during the COVID-19 simulations. Participants maintained overall good closed-loop communication, assigned roles, and shared their mental models, resulting in an improved approach to the management as simulations went on. Teams were quick at suspending disbelief, more so during the COVID simulations than any other, because of the general anxiety that comes with caring for these patients and the eagerness to learn and to do the right thing. Healthcare professionals should be viewed as assets that are integral to growth and development, offering resilience and flexibility [24]. Guiding healthcare professionals in attaining desired levels of comfort and preparedness, individually and in a team setting, helps them maintain and perpetuate the efficient practices performed/acquired during simulation, thus allowing them to become active and proactive players in a dynamic system that is greater than the sum of its parts. Resource allocation was particularly challenging because of the new space adopted for COVID-19 patients, new and fluid guidelines regarding the approach to the management of these patients, and non-native teams occasionally working together. This gives insight into the system’s resilience if we assess it through Hollnagel’s Resilience Assessment Grid (RAG); that is the measure of how well a system is able to respond, monitor, learn, and anticipate—the four crucial abilities necessary for resilient performance [24]. Our teams are better equipped to respond to COVID-19 cases and are aware of the important signs that need to be monitored. Furthermore, the debriefing sessions gave the participants the time to learn from their and others’ mistakes. The latter three points allow the team to know what to anticipate when managing a COVID-19 case. Healthcare workers in Lebanon witnessed the COVID crisis in other countries before it got to Lebanon. They quickly acquired the knowledge, adapted the environment, and embarked on aggressive simulation training of all the teams involved. This optimized the resilience of the system despite limited resources and political and financial collapse outside the walls of healthcare institutions. We have reason to believe that the system has the potential to stay resilient through further simulations of COVID-19 and other scenarios as long as we continue to monitor, learn, anticipate, and respond.

## Conclusion

We do not believe that the epidemic in Lebanon has resolved yet, and we have been successful at flattening the curve while preparing our staff and hospital to deal with this challenge. The SARS-CoV-2 virus is likely to stay with us for a while, and maintaining simulation training will assure safety and excellence in providing care. Looking at the challenges from a safety II approach will allow us to use what we do well in the most effective and efficient way to address those challenges and adapt to new guidelines so we can come out of this crisis with minimal damage.

## Supplementary information

**Additional file 1.** SET-M Results (N=131).

## Data Availability

The datasets generated and/or analyzed during the current study are not publicly available due the fact that this data pertains to internal institutional quality improvement initiatives, and the raw data and materials have not been approved for public sharing but could be available from the corresponding author on reasonable request.

## Abbreviations

COVID-19: Coronavirus disease 2019
LST: Latent safety threat
AUBMC: American University of Beirut Medical Center
PEARLS: Promoting Excellence and Reflective Learning in Simulation
SET-M: Simulation effectiveness tool-modified
PPE: Personal protective equipment
SARS: Severe acute respiratory syndrome
TST: Tetherless simulator technology
IPC: Infection prevention and control
SARI: Severe acute respiratory infection
RSI: Rapid sequence intubation
MDI: Metered-dose inhaler
CRM: Crisis resource management
DAWSIM: Dar Al-Wafaa Simulation in Medicine
