# Supplementary material for: In-situ simulations for COVID-19: a safety II approach towards resilient performance
Source: Adv Simul (Lond). 2020 Jul 29;5:15. doi: 10.1186/s41077-020-00137-x (PMC7388429; doi:10.1186/s41077-020-00137-x)
Supplement: Supplementary file 1 — Additional file 1. SET-M Results (N=131). [file 41077_2020_137_MOESM1_ESM.docx]

**SET-M Results (N=131)**

| **PREBRIEFING:** | **Strongly Agree n (%)** | **Somewhat Agree n (%)** | **Do Not Agree n (%)** |
| --- | --- | --- | --- |
| Prebriefing increased my confidence | 109 (83%) | 20 (15%) | 2 (2%) |
| Prebriefing was beneficial to my learning. | 115 (87%) | 14 (11%) | 2 (2%) |
| **SCENARIO:** | | | |
| I am better prepared to respond to changes in my patient’s condition. | 102 (78%) | 26 (20%) | 3 (2%) |
| I developed a better understanding of the pathophysiology. | 102 (78%) | 26 (20%) | 3 (2%) |
| I am more confident of my assessment skills. | 104 (79.5%) | 24 (18.5%) | 3 (2%) |
| I felt empowered to make clinical decisions. | 102 (78%) | 25 (19%) | 4 (3%) |
| I developed a better understanding of medications. (Leave blank if no medications in scenario) | 100 (76%) | 26 (20%) | 5 (4%) |
| I had the opportunity to practice my clinical decision-making skills. | 106 (81%) | 21 (16%) | 4 (3%) |
| I am more confident in my ability to prioritize care and interventions | 109 (83%) | 19 (14) | 3 (3%) |
| I am more confident in communicating with my patient. | 109 (83%) | 19 (14) | 3 (3%) |
| I am more confident in my ability to teach patients about their illness and interventions. | 103 (79%) | 25 (19%) | 3 (2%) |
| I am more confident in my ability to report information to health care team. | 106 (81%) | 22 (17%) | 3 (2%) |
| I am more confident in providing interventions that foster patient safety. | 107 (82%) | 21 (16%) | 3 (2%) |
| I am more confident in using evidence-based practice to provide care. | 109 (83%) | 18 (14%) | 4 (3%) |
| **DEBRIEFING:** | | | |
| Debriefing contributed to my learning. | 110 (84%) | 19 (14%) | 2 (2%) |
| Debriefing allowed me to verbalize my feelings before focusing on the scenario | 107 (82%) | 22 (17%) | 2 (2%) |
| Debriefing was valuable in helping me improve my clinical judgment. | 108 (82%) | 21 (16%) | 2 (2%) |
| Debriefing provided opportunities to self-reflect on my performance during simulation. | 109 (83%) | 20 (15%) | 2 (2%) |
| Debriefing was a constructive evaluation of the simulation. | 110 (84%) | 19 (14%) | 2 (2%) |
